# The Dual Effect of Ionic Liquid Pretreatment on the Eucalyptus Kraft Pulp during Oxygen Delignification Process

**DOI:** 10.3390/polym13101600

**Published:** 2021-05-15

**Authors:** Letian Qi, Jinke Liu, Jianmin Peng, Guihua Yang, Fengfeng Li, Yu Xue, Jiachuan Chen

**Affiliations:** State Key Laboratory of Biobased Material and Green Papermaking, Qilu University of Technology, Shandong Academy of Sciences, Jinan 250353, China; lqi01@qlu.edu.cn (L.Q.); ljk810319@gmail.com (J.L.); pjmdyx2018@163.com (J.P.); lifengfeng@qlu.edu.cn (F.L.); xueyu@qlu.edu.cn (Y.X.); chenjc@qlu.edu.cn (J.C.)

**Keywords:** ionic liquid, pretreatment, oxygen delignification, fiber protection

## Abstract

Oxygen delignification presents high efficiency but causes damage to cellulose, therefore leading to an undesired loss in pulp strength. The effect of ionic liquid pretreatment of [BMIM][HSO_4_] and [TEA][HSO_4_] on oxygen delignification of the eucalyptus kraft pulp was investigated at 10% IL loading and 10% pulp consistency, after which composition analysis, pulp and fiber characterizations, and the mechanism of lignin degradation were carried out. A possible dual effect of enhancing delignification and protecting fibers from oxidation damage occurred simultaneously. The proposed [TEA][HSO_4_] pretreatment facilitated lignin removal in oxygen delignification and provided fibers with improved DP, fiber length and width, and curl index, resulting in the enhanced physical strength of pulp. Particularly, its folding endurance improved by 110%. An unusual brightness reduction was identified, followed by detailed characterization on the pulps and extracted lignin with FTIR, UV, XPS, and HSQC. It was proposed that [TEA][HSO_4_] catalyzed the cleavage of β-O-4 bonds in lignin during the oxygen delignification, with the formation of Hibbert’s ketones and quinonoid compounds. The decomposed lignin dissolved and migrated to the fiber surface, where they facilitated the access of the oxidation agent and protected the fiber framework from oxidation damage. Therefore, it was concluded that ionic liquid pretreatment has a dual effect on oxygen delignification.

## 1. Introduction

Oxygen delignification is widely adopted in the pulp and papermaking process, biorefinery industry, as well as the production of value-added bio-based products for its high efficiency, low cost, and environmental protection features. Despite the complex aryl-ether bonds and carbon-carbon linkages in lignin aromatic units [1], the reactive oxygen in its aqueous solution attracts the aromatic units [2] and induces the formation of phenolic radicals [3], thereby efficiently triggering the breakage of aryl-ether bonds and decomposing lignin into smaller fragments [4,5]. However, the effectiveness of an oxygen delignification stage is usually limited to 50% in the pulp and papermaking industry [6,7], otherwise, a severe decomposition of carbohydrates occurs with undesired viscosity deterioration along with the physical strength loss of pulp [8,9]. Various pretreatment and reinforcement methods have been studied in combination with oxygen delignification to improve its overall performance [10,11,12]. For example, peroxyacid and polyoxometalate [10] treatment could increase the efficiency of oxygen delignification; however, reductions in the physical strength of paper are reported. Sodium percarbonate and hydrogen peroxide could reinforce the oxygen delignification, but they reduce pulp viscosity [11]. While the addition of sodium perborate could be employed to avoid the bleaching damage of pulp fibers, it has a minor elevation in bleachability [13]. Xylanase pretreatment that has been reported to slightly elevate the pulp viscosity, showing minor improvement in delignification efficiency [14]. Thereby, it seems difficult for a single pretreatment to simultaneously enhance the delignification and elevate the physical strength of pulp. The investigation of new techniques to improve efficiency in oxygen delignification, while preserving/improving the physical strength of pulp and paper would be attractive in both the scientific and industrial fields.

Ionic liquids (ILs) are non-volatile, stable, and recyclable solvents with good solubility and tunable features [15]. The application of ILs in the dissolution and fractionation of lignocellulosic biomass has been extensively reviewed [16,17,18]. Generally, they are widely recognized for the strong coulombic and H-bonding interactions [19], which could disturb the H-bond network of the plant fiber and facilitate lignin removal in lignocellulosic biomass [20,21]. They were widely reported for lignocellulose pretreatments [22,23] to enhance the swelling of fiber [24], changing their morphology and improving the physical strengths [25]. Interestingly, [BMIM] cation-based ILs are reported to conserve the viscosity of fiber during the pulping process [26]. Although most IL pretreatments require the absence of water for preserving their effectiveness [27], some [HSO_4_]^−^ anion-based ILs were reported recently to be effective even in their dilute aqueous solutions [28]. They were reported to efficiently disturb the cellulose-lignin-hemicellulose inter-bonds and dissolve lignin, making these ILs significant for the fractionation of the main constituents [29,30]. However, a successful design of ILs requires not only strong H-bonding abilities but also taking their viscosity, cost, and water tolerance into considerations. [TEA][HSO_4_] was reported to have low cost ($1/kg), yet with high-efficiency, fractionating plant fibers [28]. In our previous works, [HSO_4_]^−^ anion-based ILs have been applied in pretreatment, combined with various bleaching sequences, where improvements in bleachability [31] and fiber strength [32] were reported. It is uncertain as to whether [HSO_4_]^−^ anion-based ILs could provide both enhancements of delignification and fiber protection, at the same time. Therefore, a further investigation over the effect of IL pretreatment on oxygen delignification of the eucalyptus kraft pulp was thoroughly investigated in this work, with an insight into the reaction mechanism.

## 2. Materials and Methods

### 2.1. Materials

The eucalyptus wood chips were taken from a pulp mill in eastern China. 1-butyl-3-methylimidazolium hydrogen sulfate ([BMIM][HSO_4_], AR, 95%) was purchased from Shanghai Rhawn Chemical Co., Ltd. (Shanghai, China). Triethylamine (analytically reagent (AR), 99%) was purchased from Tianjin Fuyu Fine Chemical Co., Ltd. (Tianjin, China). Anhydrous magnesium sulfate (AR, 99%) and sodium hydroxide (AR, 96%) were purchased from Tianjin Da Mao Chemical Co. (Tianjin, China). Sulfuric acid (AR, 98%) was purchased from Lai Yang Chemical Co., Ltd. (Laiyang, China). Sodium sulfide (AR, 98%) was purchased from Tianjin Dingshengxin Chemical Industry Co., Ltd. (Tianjin, China).

Triethylammonium hydrogen sulfate ([TEA][HSO_4_]) was synthesized with the presence of 10 wt% water content. The detailed synthesis information was as described in the previous work [32], where a 5M H_2_SO_4_ aqueous solution was added stepwise into triethylamine with stirring.

### 2.2. Laboratory Scale Pulp Preparation

The cooking conditions for preparing kraft pulp of eucalyptus were selected according to the literature [31]: 21% of available alkali, 25% of sulfidity, 170 °C of maximum cooking temperature, and 90 min of cooking time at 170 °C. The chemical composition of the obtained kraft pulp (KP) was 81.77% of cellulose, 3.52% of hemicellulose, and 15.30% of lignin. The kappa number, brightness, and viscosity of the KP were 20.04, 28.24%ISO, and 1015 mL/g, respectively.

### 2.3. Ionic Liquid Pretreatment

The IL pretreatment was performed in a water bath, where 25 g of over-dried KP was homogeneously mixed with or without the presence of 2.5 g IL; subsequently, approximately 250 mL water was added into the sealed polyethylene bag to keep 10% of the consistency mixture. The pretreatment was performed at 60 °C, for 60 min.

### 2.4. Oxygen Delignification

Oxygen bleaching was carried out based on the literature method [32], during which the pretreated pulp was directly delignified at 0.5 MPa of oxygen pressure, 4% of Na_2_O, and 100 °C, for 60 min. The control sample was bleached at the same condition (10% pulp consistency, 0.5 MPa O_2_, 4% Na_2_O, and 100 °C, for 60 min).

### 2.5. Pulp Analysis

The content of cellulose, hemicellulose, and lignin were determined according to TAPPI standard T 201 wd-76, T 223 cm-01, and T 13 wd-74, respectively.

The viscosity was obtained using the method of ISO 10650:1999, and the degree of polymerization (DP) of pulp was calculated based on the pulp viscosity. DP was calculated according to the literature method [31,33].

Pulp yield was calculated as the mass ratio of the pulp sample before and after bleaching. The mass ratios of the pulp samples before and after treatment were calculated as pulp yield.

The kappa number of the pulp was determined according to the ISO 302:2004 method.

The water retention value (WRV) of the pulp was determined according to the ISO 23714:2013 method.

The weight mean length, weight mean width, and fines content of the pulp fibers were measured using a fiber quality analyzer (FQA-LDA02, OpTest Equipment Inc., Hawkesbury, ON, Canada).

### 2.6. Pulp Hand-Sheet Preparation

The treated pulp was dispersed with water and formed hand-sheets in grammage of 80 g/m^2^; subsequently, hand-sheets were kept in the environment, at 23 °C and 50% humidity, before testing. The tensile index, burst index, tear index, folding endurance, and brightness of the hand-sheets were measured according to the ISO methods 15754:2010, 2758:2014, 1974:2012, 5625:1997, and 2470:1999, respectively.

### 2.7. Extraction of Lignin from Kraft Pulp

Lignin was isolated from the kraft pulp according to the reported procedure [34]. Briefly, three kraft pulps were put into a ball mill, for 72 h, at room temperature. Subsequently, the powder was extracted with a dioxane–water mixture and centrifuged to obtain a supernatant. The supernatant was put onto a rotary evaporator to remove solvents and was then freeze-dried to obtain the lignin product.

### 2.8. Characterization Analysis

#### 2.8.1. Ultraviolet Spectroscopy (UV) Analysis

The quinone compounds content was measured using a Cary 8454 UV-Vis (Agilent Technologies, Santa Clara, CA, USA) analysis, with the configured sample solution of 3.5 g/L and a measuring absorbance at 450 nm.

#### 2.8.2. Scanning Electron Microscopy (SEM) Analysis

The morphology of pulp fiber was analyzed using a Regulus 8220 FE-SEM (Hitachi High-Technologies Corporation, Tokyo, Japan) operated at 5 kV accelerating voltage, with 2000 times magnification.

#### 2.8.3. X-ray Photoelectron Spectrometer (XPS) Analysis

The surface lignin content of pulp was determined using an X-ray photoelectron spectrometer (XPS), according to the literature approach [35,36], where a linear relationship between the O/C ratio and surface lignin content was applied. The calibration was done using an O/C ratio of 0.74 for pure cellulose (Whatman filter paper), and 0.33 for pure lignin. Before XPS analysis, the pulp sample of the hand-sheets was pumped in a drying oven at 60 °C, for 24 h, to eliminate residual water. The XPS analysis was carried out with Kratos Axis Ultra spectrometer, using a monochromatic AI k (alpha) source (10 Ma,15 V), probing the surface of the sample to a depth of 5–7 nm, ranging from 0.1 to 0.5 atomic percent, depending on the element. The Kratos charge neutralizer system was used on all specimens. Survey scan analysis was taken with an analysis area of 300 × 700 μm and a pass energy of 160 eV, while the high-resolution scan was also recorded with an analysis area of 300 × 700 μm and a pass energy of 20 eV. Measurements were taken at three different spots on each sample to attain an average over the heterogeneity of the samples.

#### 2.8.4. Fourier Transform Infrared Spectroscopy (FT-IR) Analysis

The Fourier transformed infrared (FT-IR) spectra of the hand-sheets and lignin were recorded using a spectrophotometer (IR Irdison-21, Shimadzu, Japan), with a resolution ratio of 4 cm^−1^, scanning speed of 32 s^−1^, and scanning range of 4000–500 cm^−1^.

#### 2.8.5. X-ray Diffraction (XRD) Analysis

The crystallinity of the pulp fiber was measured by X-ray diffraction (XRD D8-DVANCE Bruker, Germany). The measurements were conducted under the conditions of X-ray 40 kW and 35 mA, with an angle from 5 to 60°. Crystallinity was determined according to Equation (1) [31,37]:

*CrI* = 100 × (*I*_002_ − *I_am_*)/*I_am_* (1)

where, *CrI* is the crystallinity of cellulose, *I*_002_ is the scattering intensity of the diffraction of 002 plane, and *I_am_* is the diffraction intensity at 2θ = 15.6°.

#### 2.8.6. HSQC NMR Analysis

NMR (models for Bruker AVANCE III 500 MHz, Bruker, Karlsruhe, Germany) were recorded, according to the literature method [34], by using a spectrometer equipped with a DCH cryoprobe. HSQC spectra were recorded at 25 °C, using the Q-CAHSQC pulse program. Matrices of 2048 data points for the ^1^H-dimension (13 to −1 ppm) and 1024 data for the ^13^C-dimension (160 to 0 ppm) were collected, with the relaxation delay set at 6 s. The lignin samples were dissolved in 0.5 mL of dimethylsulfoxide-d6 (DMSO-d_6_), and chemical shifts were referenced to the solvent signal (2.50/40.21 ppm).

## 3. Results

### 3.1. Effect of IL Pretreatments on Pulp Bleachability

To investigate the effect of IL pretreatment on oxygen delignification, both [BMIM][HSO_4_] and [TEA][HSO_4_] pretreatments were performed at 10% IL loading based on oven-dry eucalyptus kraft pulp (KP) weight with 10% pulp consistency. Therefore, both ILs were applied within their dilute solutions (~1 wt%). The pretreated pulp was then oxygen-delignificated at 0.5 MPa O_2_, 4% Na_2_O, and 100 °C, for 60 min. The composition of KP and the oxygen-delignified pulp with or without IL pretreatments are shown in Table 1, where the cellulose content of the bleached pulp increased from 81.77% to 93.11% in the control sample, and the [BMIM][HSO_4_] and [TEA][HSO_4_] pretreated samples are 94.14% and 94.22%, respectively. Oxygen delignification efficiently decreased the lignin content of pulp, from 15.30% to 6.76%. Both IL pretreatments improved the lignin removal during the oxygen delignification stage because the lignin content in [BMIM][HSO_4_] and [TEA][HSO_4_] pretreated samples were 5.96% and 3.52%, respectively. There is no doubt that ILs used in this work efficiently facilitated the lignin degradation/dissolution, even in its diluted aqueous solutions. These findings were confirmed by the characterization of pulp showed in Table 2, where the kappa number of pulp reduced from 20.4 to 8.06 in the control sample, and further reduced to 7.47 and 7.04 in [BMIM][HSO_4_] and [TEA][HSO_4_] pretreated samples, respectively. More promisingly, a well-preserved fiber framework was observed in both IL pretreated samples. The pulp viscosity increased from 863 mL/g in the control test to 905 and 999 mL/g in [BMIM][HSO_4_] and [TEA][HSO_4_] pretreated samples, respectively. A trend of fiber protection based on the sample DP value was [TEA][HSO_4_] > [BMIM][HSO_4_]; moreover, [TEA][HSO_4_] presented an excellent fiber protection, whose DP reached 1501, almost the same as that in unbleached KP (DP = 1527); in comparison, the DP of the control sample was only 1277. In the meantime, only a minor drop in the pulp yield was viewed in both of the IL pretreated samples; this ~1% yield loss possibly matched with their synergetic lignin removal in oxygen bleaching. Therefore, both of the IL pretreatments in this work facilitated the lignin degradation/dissolution and protected the pulp fiber simultaneously; moreover, [TEA][HSO_4_] pretreatment presented an improved synergetic effect compared with that of [BMIM][HSO_4_].

Interestingly, an unexpected low brightness was viewed for both IL pretreated samples. The oxygen delignification improved the brightness from 28.24%ISO to 56.07%ISO; however, for [BMIM][HSO_4_] and [TEA][HSO_4_] pretreated samples, the brightness was only 54.27 and 42.28%ISO, respectively. The unusual brightness reduction in both [HSO_4_]^−^ anion-based ILs disagreed with their low lignin content that was reported earlier in this work. It also conflicted with our previous finding [32], where an improvement in bleachability, including brightness, was reported for similar IL pretreatments in a more complete ODP bleaching sequence. A possible hypothesis was that these [HSO_4_]^−^ anion based ILs not only facilitated lignin degradation/dissolution in the oxygen delignification process, but also catalyzed the formation of Hibbert′s ketones [39,40], which could be converted into quinonoid compounds [41] in aqueous solutions, resulting in a brightness reduction [42]. These quinonoid compounds could only be the intermediate chemicals and be easily consumed in the subsequent peroxide bleaching stages, thereby presenting no influence on the final brightness of the pulp at the end of the bleaching sequences. The formation of quinonoid compounds was confirmed by UV absorbance at 450 nm [38], where the absorbance increased from 0.212 in the control sample to 0.269 and 0.334 in [BMIM][HSO_4_] and [TEA][HSO_4_] pretreated samples, respectively. The [TEA][HSO_4_] pretreated sample presented a lower overall lignin content and a higher quinone content than those of [BMIM][HSO_4_] samples, illustrating the smaller cation and less shielded positive charge in IL, which facilitated the lignin removal and decompositions in the oxygen delignification process.

### 3.2. Effect of ILs Pretreatment on Pulp Properties

The properties of the pulp with or without IL pretreatments during the bleaching process are shown in Table 3. The oxygen delignification caused a decrease in the fiber length and width; however, both IL pretreatments protected the pulp fibers from degradation. Especially for the sample pretreated by [TEA][HSO_4_], only minor changes in fiber length and width could be viewed. Its number-averaged fiber length (*Lc(n)*) only dropped from 0.679 to 0.670 mm, and fiber width only dropped from 14.29 to 13.94 μm. This is while the *Lc(n)* and width of the control sample were only 0.647 mm and 13.46 μm, respectively. Meanwhile, the curl index increased from 9.12% to 14.48% for the control pulp, and it further improved to 14.81% and 15.66% in [BMIM][HSO_4_] and [TEA][HSO_4_] pretreated samples, respectively. The well-preserved fiber framework and the increase in curl index enhanced the physical strength properties of the pulps [43]. The improvement in physical strength is clearly viewed in Table 4, where the tensile index, tear index, and, especially, the folding endurance of the IL pretreated samples were all improved. The tensile index improved from 73.25 in the control sample to 75.50 and 81.00 N·m·g^−1^ in [BMIM][HSO_4_] and [TEA][HSO_4_] pretreated samples, respectively. Similarly, tear index in [BMIM][HSO_4_] and [TEA][HSO_4_] pretreated samples improved from 8.47 to 9.32 and 9.81 mN·m^2^·g^−1^, respectively. Particularly, the folding endurance of hand-sheets pretreated by [BMIM][HSO_4_] and [TEA][HSO_4_] increased by 66% and 110%, respectively, compared with the control sample. Although the fiber width data showed minor difference, a clear trend of [TEA][HSO_4_] > [BMIM][HSO_4_] > control test was noticed. It is highly possible that [TEA] cation with stronger cationic charge and smaller molecular size could form stronger/more interaction with water to generate a larger IL-water cluster than that of [BMIM][HSO_4_]. During the pretreatment, the IL-water cluster interacted with pulp fiber majorly through H-bonding. As they entered the amorphous region of the fiber, the [TEA][HSO_4_] with a larger hydrated cluster caused a more swelled fiber than [BMIM][HSO_4_], easing the lignin dissolution in the bleaching process thereafter.

The pulp characterization results supported those of the previous finding, where IL pretreatments not only enhanced the lignin degradation and removal but also protected the pulp fiber from degradation during the oxygen delignification process. Taking [TEA][HSO_4_] pretreated bleached pulp, for example, its viscosity, DP, and fiber length, were all similar to those in the original KP; in comparison, all the mentioned parameters significantly decreased in the control sample. This indicated that the severe degradation of pulp fibers in oxygen delignification could be avoided with the [TEA][HSO_4_] pretreatment and their presence in oxygen delignification. In the meantime, lignin removal was clearly improved in [TEA][HSO_4_] samples, as the lignin content and kappa number decreased significantly, together with improvements in curl index and water retention values. All these merits consequently contributed to an enhancement in the physical strength of pulp hand-sheets [44].

### 3.3. Pulp Fiber Surface Morphology and Characterization

**Figure 1:**
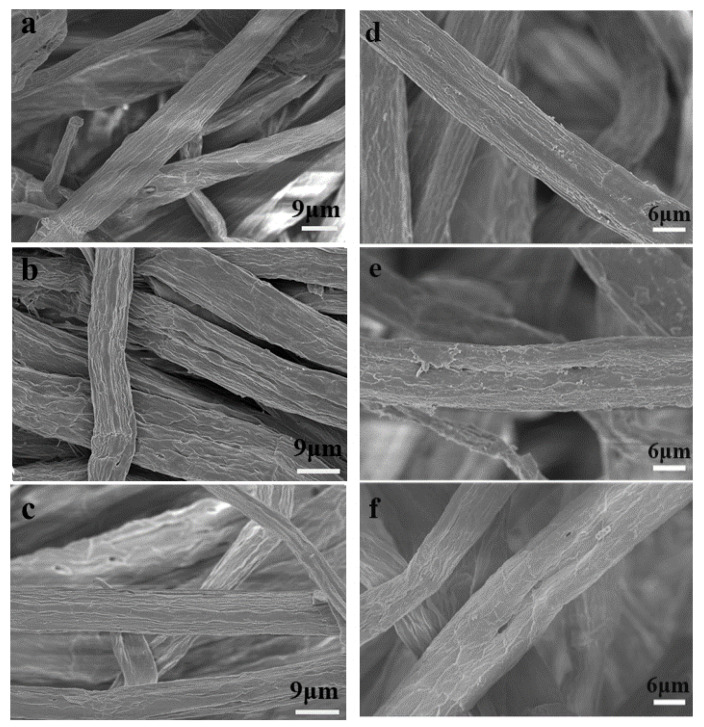
SEM images of oxygen-delignificated kraft pulps with or without IL pretreatments: (**a**) control pulp, ×1800; (**b**) [BMIM][HSO_4_] pretreated pulp, ×1800; (**c**) [TEA][HSO_4_] pretreated pulp, ×1800; (**d**) control pulp ×2000; (**e**) [BMIM][HSO_4_] pretreated pulp, ×2000; and (**f**) [TEA][HSO_4_] pretreated pulp ×2000.

**Figure 2:**
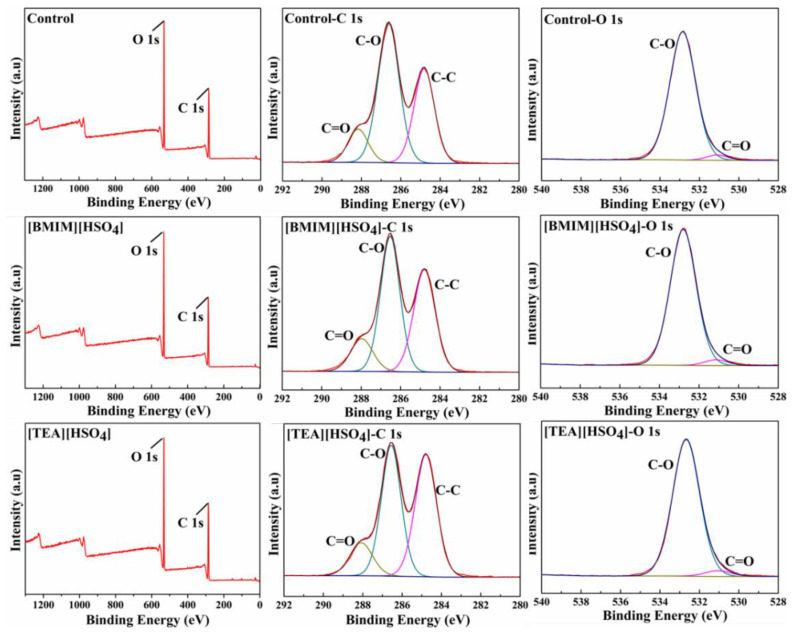
XPS analysis of oxygen-delignificated kraft pulps with or without IL pretreatments.

The morphology of the KP fibers after oxygen bleaching with or without Il pretreatments are shown in Figure 1, where the fibers of control pulp (a) show a relatively smooth surface, while the [BMIM][HSO_4_] (b) and [TEA][HSO_4_] (c) pretreated samples present a more roughed fiber surface, but the fiber framework is well preserved under the same magnification. Further magnification on fiber surface are shown in Figure 1d–f, where cracks and holes on fiber surface could be noticed, which might prove the successful lignin removal from the pulp. Some sticky small fragments were identified in [BMIM][HSO_4_] (e) and [TEA][HSO_4_] (f) pretreated samples, which might be generated from the dissolved lignin migrating to the fibers’ surface. The lignin deposited on the surface of pulp fibers was detected by XPS spectrums, with results shown in Figure 2. As expected, the surface elements were mainly O and C. The ratio of oxygen to carbon atoms (O/C) on the fiber surface was calculated by the sensitivity factor of the element, which was further applied for the determination of surface lignin content. It was reported that the O/C ratio of pure cellulose (Whatman filter paper) was 0.74, and the O/C ratio of pure lignin was 0.33 [35,36]. From Figure 2, the O/C ratio of the control, [BMIM][HSO_4_], and [TEA][HSO_4_] pretreated samples are 0.53, 0.51, and 0.48, respectively. Therefore, the surface lignin content of the control, [BMIM][HSO_4_] and [TEA][HSO_4_] pretreated samples are 51.22%, 56.10%, and 63.42%, respectively, which clearly demonstrated the increased surface lignin content in IL pretreated pulp samples. The [TEA][HSO_4_] pretreated sample presented a lower overall lignin content, a higher quinone content, and a more fiber surface lignin content, compared with the [BMIM][HSO_4_] pretreated sample. The reason may be that the [TEA][HSO_4_] cation presented a higher affinity with the anionic on the surface of the pulp fiber due to its size and charge distribution, which promoted its contact with lignin and catalyzed the degradation of lignin into fragments for dissolution, therefore presenting an improved lignin decomposition compared with that of [BMIM] cation based IL.

It was interesting to notice that the ILs pretreatment dissolved lignin from pulp fiber, decreasing the overall lignin content, while increasing the lignin content on the pulp fiber surface after oxygen delignification. The deposited lignin facilitated the preferential access of the bleaching agent to the lignin, thus improving the delignification efficiency of the bleaching agents and protecting the cellulose framework from oxidation damage.

### 3.4. Characterization of Oxygen Bleached Fibers

**Figure 3:**
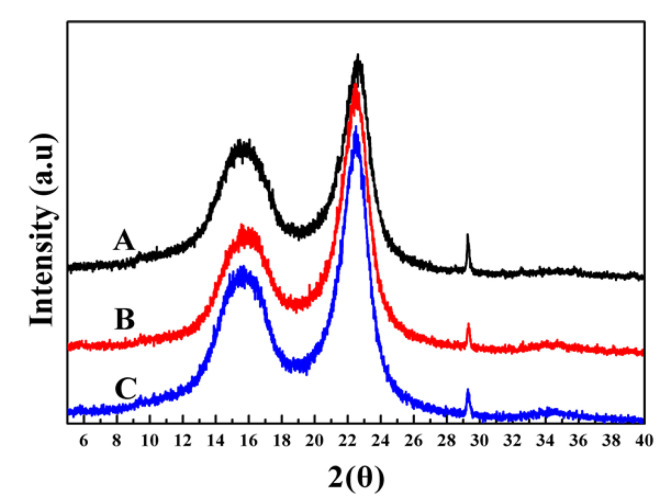
XRD analysis of oxygen-delignificated kraft pulps with or without IL pretreatments: (A) control pulp, (B) [BMIM][HSO_4_] pretreated pulp, and (C) [TEA][HSO_4_] pretreated pulp.

XRD analysis of fibers shown in Figure 3, where cellulose fractions presented two characteristic peaks, at 15.6° (*I_am_*) and 22° (*I*_002_), respectively, indicate a well-preserved natural cellulosic structure. No new characteristic peaks were identified, except for the changes in intensities. The crystallinity of the control pulp and that of the pulp pretreated by [BMIM][HSO_4_] and [TEA][HSO_4_] after the oxygen delignification process were 45.8%, 57.9%, and 58.3%, respectively. The IL pretreatments significantly improved the crystallinity of the pulp, especially in that of [TEA][HSO_4_] pretreated sample, where crystallinity increased by 12.5%, compared with the control pulp. These results are in line with improvement in the fiber quality and physical strength of pulp hand-sheet, thus confirming a well-preserved cellulosic framework in IL pretreated pulps. In comparison with [BMIM][HSO_4_] pretreated sample, the higher crystallinity observed in [TEA][HSO_4_] pretreated sample indicates that they can interact more efficiently with the amorphous region of the fiber, improving the swelling and also the lignin dissolution.

**Figure 4:**
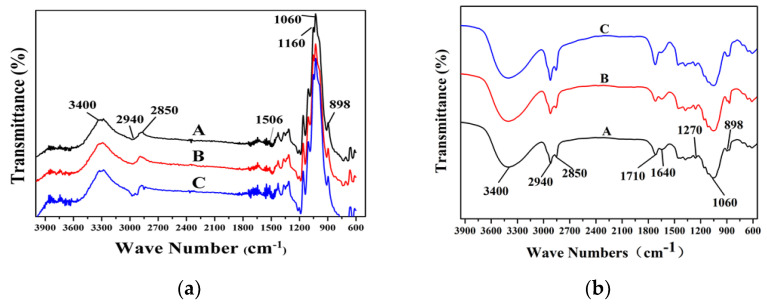
FT-IR spectra of oxygen-delignificated kraft pulps and the extracted lignin with or without IL pretreatments: (**a**) FT-IR spectra of oxygen-delignificated pulps: (A) control pulp, (B) [BMIM][HSO_4_] pretreated pulp, and (C) [TEA][HSO_4_] pretreated pulp; (**b**) FT-IR spectra of lignin extracted from oxygen-delignificated pulps: (A) lignin in control pulp, (B) lignin in [BMIM][HSO_4_] pretreated pulp, and (C) lignin in [TEA][HSO_4_] pretreated pulp.

The FT-IR analysis of the chemical structure of the bleached pulps are shown in Figure 4a, with the assignment of characteristic peaks listed in Table 5. The absorption peak at 3400 cm^−1^ was derived from -OH stretching vibration, and the absorption peak at 2940–2850 cm^−1^ was derived from C-H stretching vibration, while 1437 cm^−1^ and 1335 cm^−1^ represented the characteristic absorption peak of lignin [32,45]. The absorption peak at 1160 cm^−1^ is C-O-C bending vibration, and the absorption peak at 1060 cm^−1^ is related to C-O-C tensile vibration. The absorption peak at 898 cm^−1^ is a β-type glycosidic bond, indicating that the xylan units were linked by a β-type glycosidic bond in the bleached fibers [46]. Therefore, we illustrated a possible formation of lignin-carbohydrate complex (LCC) connections between lignin and carbohydrates. Generally, no new characteristic peak of the pretreated pulp appeared, except the intensity variations. Therefore, ILs pretreatment did not result in major changes of functional group and chemical compositions of pulp fibers.

However, due to the low lignin content in bleached pulp fibers, it was difficult to analyze the structural information on the lignin component. Figure 4b shows the characterizations of lignin extracted from oxygen-delignificated pulps, removing the interface of carbohydrate shown on the FT-IR results. Typical lignin benzene ring skeleton bands were found at 1640 cm^−1^ in all testing samples, illustrating the presence of lignin framework after oxygen delignification. A typical GS type of lignin could be viewed in all testing samples, as the absorption bands at 1115 cm^−1^ corresponded to C–H stretching vibration in syringyl units, while the absorbance at 1270 cm^−1^ corresponded to C–O stretching vibration of the guaiacyl units. The absorption band at 1710 cm^−1^ was corresponded to the unconjugated C = O stretching vibration, where a significant increase of intensity was viewed in IL pretreated samples, especially in the [TEA][HSO_4_] pretreated sample. Therefore, a formation of ketones was confirmed, and it is obvious that [TEA][HSO_4_] pretreated sample generated more ketone-based fragments. However, the signals of carbohydrate bands at 1160, 1060, and 898 cm^−1^ were identified clearly in all samples, which indicated the presence of carbohydrate impurities in the extracted lignin sample. The lignin was extracted from the bleached pulp by 72 h ball-mill, followed by a dioxane-water extraction and freeze-dry that should efficiently remove the carbohydrate fractions. A possible reason for the presence of impurity would be the formation of LCC during the oxygen delignification, in which the degradation and dissolution of lignin occurred in combination with the formation of LCC between lignin and carbohydrates.

Based on the characterization and analysis, a dual effect of IL pretreatment in the oxygen delignification process was proposed, where the [HSO_4_]^−^ based ILs, especially the [TEA][HSO_4_], could efficiently facilitate the lignin degradation/dissolution in oxygen delignification, which significantly reduced the overall lignin content, providing pulp with improved bleachability. During the process, it also acted as a catalyst to accelerate the oxygen delignification on the fiber surface, where the lignin macromolecules were degraded with the cleavage of β-O-4 bonds, with the formation of ketones and thereafter quinonoid compounds [39,40,41]. The degraded lignin dissolved and migrated/deposited on the pulp fiber surface and formed LCC with carbohydrates thereafter. The deposited lignin was preferably accessed by an oxidation agent, thereby protecting pulp fiber from oxidation damage during the oxygen delignification process. The formation of quinonoid compounds reduced the pulp brightness. However, these quinonoid compounds could only be intermediate and be easily destructed by peroxide in the subsequent bleaching stages [32], thereby presenting no influence on the brightness of the final pulp at the end of the bleaching sequences.

### 3.5. HSQC Analysis

**Figure 5:**
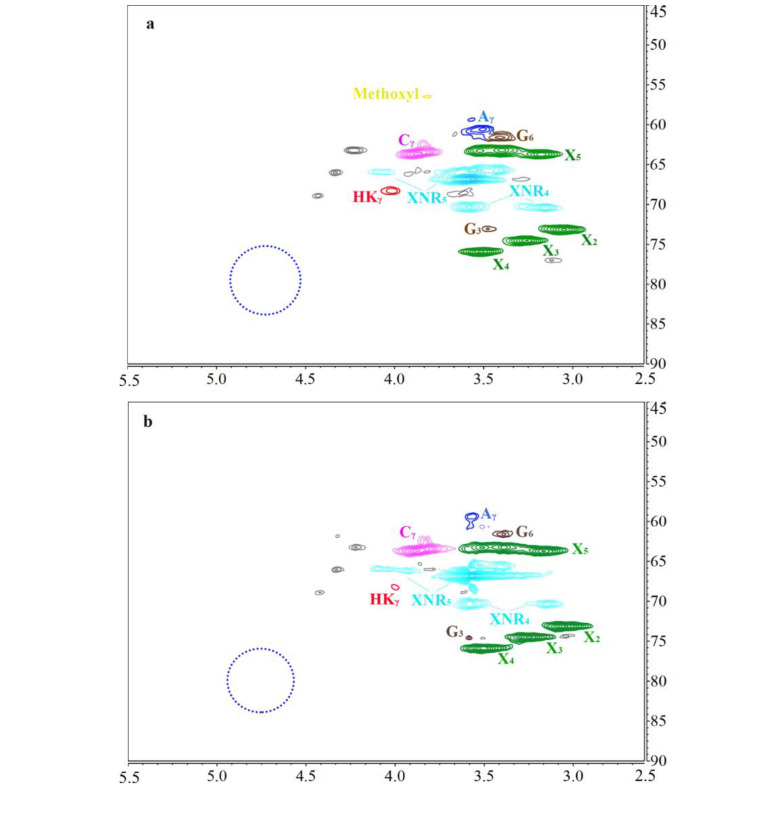

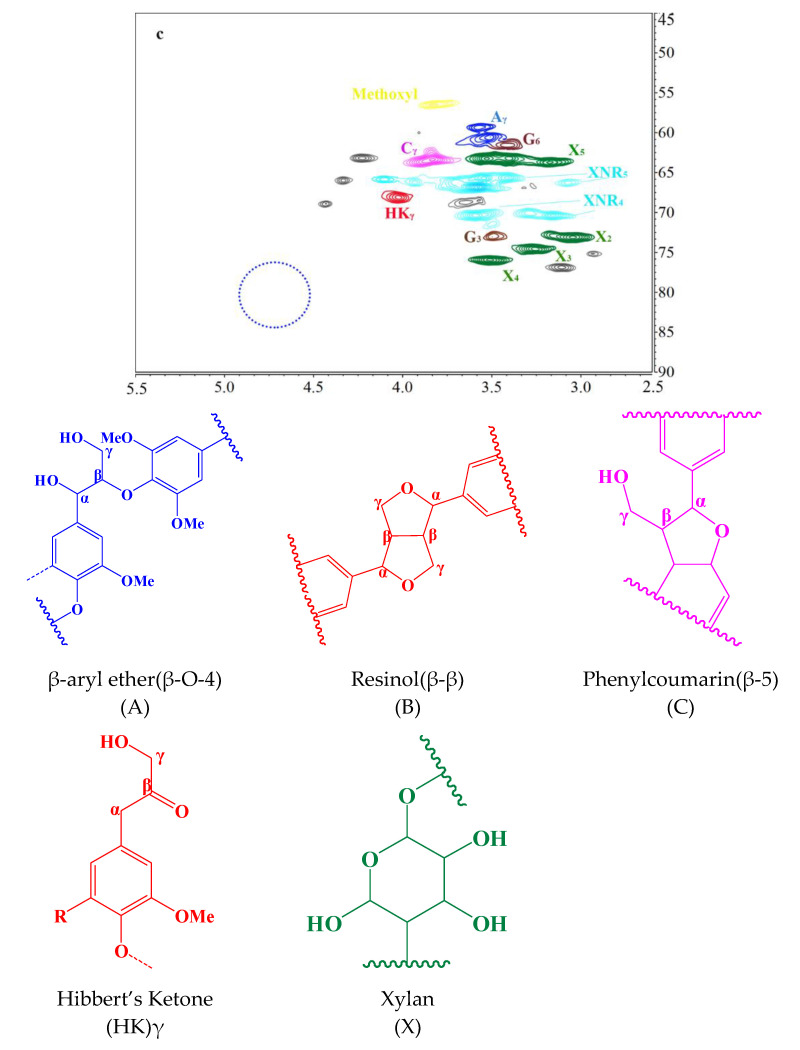
HSQC analysis of the oxygen-delignificated pulp lignin with or without IL pretreatment: (**a**) lignin in control pulp, (**b**) lignin in [BMIM] [HSO_4]_ pretreated pulp, and (**c**) lignin in [TEA] [HSO_4_] pretreated pulp. (A) β-O-4 ether bond; (B) β-β resinol bond; (C) β-5 phenylcoumarin bond; (HK) Hibbert’s ketone; (X) xylan.

Further characterizations with lignin side chains are shown in Figure 5, in which the assignments of the peaks in the spectra are based on a previous report [34]. The peaks corresponding to β-aryl ether connections are shown for Aα (δC/δH 4.95/69.92 ppm) and Aγ (δC/δH 3.51/61.69 ppm). β-β connections are shown for Bα (δC/δH 4.70/85.49 ppm), Bβ (δC/δH 3.06/53.71 ppm), and Bγ (δC/δH 3.86/71.80 ppm). β-5 connections are shown for Cα (δC/δH 5.59/87.55 ppm), Cβ(δC/δH 3.41/52.53), and Cγ(δC/δH 3.89/63.69 ppm). The absence of typical β-O-4 bonds (blue cycle, δC/δH 4.25-4.80/77.00-83.00 ppm) could be seen in all the samples, which confirm successful cleavage β-O-4 bonds in lignin macromolecules. A typical Hibbert’s ketone (HK) structure (HKγ, δC/δH 4.02/68.09 ppm) was observed in the samples, indicating a conversion of the lignin fragments to Hibbert’s ketone. It was reported that these HKs generated from lignin decomposition could be converted into quinonoid compounds [41], resulting in a brightness decrease [42]. As shown in Figure 5c, more HK structures were observed in [TEA][HSO_4_] pretreated sample, which is consistent with its brightness reduction, showed in Table 2. The low content of methoxy connections was shown at (δC/δH 3.76/56.33 ppm) in all testing samples, which indicated a possible presence of impurities affecting the lignin peak intensities. In the meantime, these lignin samples presented various LCC connections with carbohydrates in the pulp (X, δC/δH 3.05–3.51/73.14–75.91 ppm and δC/δH 3.17–3.40/63.20–63.73 ppm), which confirm the formation of LCC in oxygen delignification. The formation of HK and LCC compounds validates the proposed delignification mechanism of IL. Therefore, these characterization results support the hypothesis of the dual effect of IL pretreatments on the eucalyptus kraft pulp during the oxygen delignification process.

As both ILs are composed by [HSO_4_]^−^ anion, the present work demonstrated the importance and effectiveness of the cations on the bleaching process. Generally, [TEA][HSO_4_] pretreated samples presented a higher delignification efficiency and better-preserved pulp fibers than those in [BMIM][HSO_4_] pretreated samples. Considering the anionic nature of KP fibers, they present a high affinity towards cationic chemicals [47]. [TEA][HSO_4_] has a smaller cation and a less shielded positive charge, which would interact with fiber surface more closely to interrupt the H-bond network of lignocellulosic macromolecules, thereby presenting an improved lignin dissolution compared with those in conventional [BMIM][HSO_4_] pretreated samples. Meanwhile, the close interaction allows [TEA][HSO_4_] to work as a more effective catalyst for lignin depolymerization, cleaving the β-O-4 linkages with the formation of Hibbert’s ketones and quinonoid compounds. Therefore, the [TEA][HSO_4_] achieved an improved performance in the decomposition and dilution of lignin macromolecules. The remaining lignin fragment migrated onto the fiber surface, where they interacted with carbohydrates and formed LCC. Although the surface lignin and the quinonoid compounds temporally reduced the brightness, they were preferably accessed by oxidation agents, thereby protecting pulp fiber from oxidation damage. Thus, a promising dual effect of [TEA][HSO_4_] pretreatment in oxygen delignification was identified in this work, where the IL not only promoted delignification but also protected the fibers from bleaching damages.

## 4. Conclusions

The dual effect of [HSO_4_]^−^ anion-based IL pretreatments on oxygen delignification was obtained, where both the enhancement of delignification and the protection of fibers occurred simultaneously in the delignification process. [TEA][HSO_4_] with stronger cationic charge and smaller size, which could form stronger/more interactions with fiber and water molecules, is preferred. The proposed IL pretreatment efficiently promoted the lignin degradation/dissolution to provide pulp with low lignin content and a well-preserved fiber framework, thereby providing pulp with improved physical strength. A possible lignin degraded/decomposition mechanism with the presence of IL was proposed based on the characterization of pulp and extracted lignin. ILs catalyzed the cleavage of β-O-4 bonds of lignin, with the formation of Hibbert’s ketones and quinonoid compounds. The degraded/decomposed lignin dissolved and migrated/were deposited to the pulp fiber surface. The deposited lignin was preferably accessed by an oxidation agent, thereby protecting pulp fiber from oxidation damage during the oxygen delignification process, which can be easily destructed in the subsequent bleaching stages.

## Figures and Tables

**Table 1:** The effect of ILs pretreatment on the chemical compositions of eucalyptus kraft pulps during the oxygen delignification process (wt%, based on over-dry pulp weight).

Pulp	Cellulose	Hemicellulose	Lignin
KP	81.77 ± 0.15	3.52 ± 0.06	15.30 ± 0.22
Control	93.11 ± 0.09	1.28 ± 0.07	6.76 ± 0.21
[BMIM][HSO_4_]	94.14 ± 0.17	0.52 ± 0.10	5.96 ± 0.15
[TEA][HSO_4_]	94.22 ± 0.11	2.05 ± 0.11	3.52 ± 0.17

**Table 2:** The effect of ILs pretreatment on the properties of eucalyptus kraft pulps in the oxygen delignification process.

Pulp	Brightness (%ISO)	Kappa Number	Viscosity (mL/g)	DP	Pulp Yield (%)	WRV (g/g)	Quinone Compounds (D_450_) ^1^
KP	28.24 ± 0.20	20.04 ± 0.08	1015 ± 3	1527	100.0	1.32 ± 0.02	--
Control	56.07 ± 0.22	8.06 ± 0.08	863 ± 2	1277	99.3	1.46 ± 0.07	0.212
[BMIM][HSO_4_]	54.27 ± 0.17	7.47 ± 0.08	905 ± 4	1346	98.6	1.45 ± 0.03	0.269
[TEA][HSO_4_]	42.28 ± 0.09	7.04 ± 0.08	999 ± 3	1501	98.2	1.62 ± 0.02	0.334

^1^ Quinone compounds content determined UV absorbance at 450 nm [38].

**Table 3:** The effect of ILs pretreatment on the fiber qualities of eucalyptus kraft pulps during the oxygen delignification process.

Pulps	Fiber Length (mm)		Fiber Width (μm)	Curl Index (%)	Fines Content (%)
	*Lc(n)*	Lc(w)			
KP	0.679	0.858	14.29	9.12	7.57
Control	0.647	0.800	13.46	14.48	6.02
[BMIM][HSO_4_]	0.666	0.840	13.79	14.81	5.99
[TEA][HSO_4_]	0.670	0.829	13.94	15.66	6.28

**Table 4:** The effect of IL pretreatment on the physical strength of oxygen-bleached hand-sheet.

Pulps	Tensile Index (N·m·g^−1^)	Tear Index (mN·m^2^·g^−1^)	Folding Endurance (times)
Control	73.25 ± 0.13	8.47 ± 0.19	166 ± 2
[BMIM][HSO_4_]	75.50 ± 0.38	9.32 ± 0.09	275 ± 4
[TEA][HSO_4_]	81.00 ± 0.38	9.81 ± 0.21	348 ± 3

**Table 5:** Assignment of FT-IR spectra of oxygen-delignificated kraft pulps and the extracted lignin with or without IL pretreatments.

Wavenumbers (cm^−1^)	Assignment
3400	O–H stretching vibration
2940, 2850	C–H stretching vibration in methyl
1710	unconjugated C = O stretching vibration
1640	Aromatic ring skeleton vibration
1270	C–O stretching vibration of guaiacyl units
1050	C–H bending vibration of guaiacyl units
898	β-type glycosidic bond

## Data Availability

All data used during the study appear in the submitted article.
